# Correspondence

**Published:** 1874-11

**Authors:** F. H. Rehwinkle


### Correspondence,

EDITOR DENTAL REGISTER:

In the October No. of the DENTAL REGISTER, page 417, appears an article, headed: "Improvement in working Rubber," by Luis Dunbar, D. D. S.

The article is so full of erroneous statements, and evinces such a want of correct information on the true and real points of the Cummings (not "Cummins") patent, that a correction is needed, lest some unfortunates, equally innocent of knowledge on this subject, may be led into trouble.

The writer starts out with questioning the occasion for any "talk about the Rubber Company's monopoly, and then informs us, that notwithstanding "a volume of enormous proportions" has been written on the subject of rubber litigation, and elaborate arguments made to show the falsity of the company's claims upon the profession, no good has resulted; and that the company recently obtained three decisions in their favor.

The last part of this statement is true as far as it goes.

The writer, Dr. D., says further: "It has long been conceded that the Cummin's claim is not for the application of rubber for dental purposes, but for the method of working it as specified in his re-issue, No. 1904."

Of all reckless and ill-conceived statements, this certainly takes the lead! Was Dr. Dunbar in the full possession of his mental faculties when he made it? I doubt it! For he certainly proves that he is attempting to speak knowingly about a subject which he does not seem to understand nor comprehend in its simplest point.

It is to be deplored that the Dr's views are erroneous,for if they were correct, there would not remain the shadow of a claim against the dentists.

Does he not know that according to Judge Shepley's decree, all claims as to methods of making vulcanite plates, moulds, process or manner of hardening rubber, were lost by the plaintiffs and gained by the defendants? Has no good then issued from litigation.

The present state of the case may be summed up in a few words, and is as follows: The Goodyear Dental Vulcanite Company, in the suit against Daniel H. Smith, were beaten in the claims as to the manner or method of working rubber, moulds and everything else were given up, and the only point on which the patent was sustained, was the question, whether a set of teeth made of vulcanized rubber was a new product or manufacture, or merely the substitution of one known material for another. The court decided that a set of teeth mounted on rubber, commonly called "vulcanite' was a new manufacture.

Many eminent, and equally well-learned lawyers differ on this question with Judge Shepley, they being of the former opinion.

The whole question, henceforth, involved in this interesting litigation is one of law, viz: Is a vulcanite set of teeth a new manufacture, or is it not?

No abjudication of this question by any tribunal except that of the U. S. Supreme Court, can, or will be final.

The case is now stripped of all side issues and all other qustions, and in this shape is awaiting its turn on the calender of the Supreme Court of the United States.

Dr. Dunbar says further: "That this (the nature of the Cumming's claim) has been talked of and explained at dental meetings over and over again, and improvements suggested, etc.

Whilst it is true that this chronic rubber litigation has been talked of and explained at our dental meetings, never, to my knowledge, has any one who pretended to know anything about the matter explained it in accordance with the views of Dr. Dunbar, nor talked about it in the manner in which he does.

After a modest rebuke to the whole dental profession for its slowness in adopting this talked of improvement (?) instead of sticking to the ruts of old time practice as far as rubber is concerned, and passing a verdict adverse to all the improvements without ever trying them, the doctor goes on to describe his improvement. Now I have no fault to find with him for doing this. He has done it well and comprehensively. I ask for pardon if I fail to see any thing new or novel in it; neither will I say aught for or against the practicability of this process. Cummings did not find it practicable, for it was substantially his process, and the sole cause of the abandonment of his claimed invention from 1855 to 1864. Cumming's describes this identical manner of making vulcanite rubber in his first application for a patent which was rejected, 1855. In the mean time, Mr. Hearing files his application, and in his drawings and specifications appear the moulds. His application for a patent is also rejected, and the whole matter rests. Drs. Putnam, Franklin and others obtained the first practicable results and improved the process of working the rubber and brought the flasks containing the moulds into use. Then! not before, Cummings and others became aware that a set of teeth for practicable purposes could be made by the improved process.

Cummings was instructed how to use the moulds, and may be used them ever afterward. In 1864, however, he makes a new application for a patent upon the old specification, describing again the process of Dr. Dunbar's improvement. No mention is made of any moulds or other process, and obtains a patent therefore. See patent No. 43,009.

This process is more fully described by Mr. Caleb Williams, in a paper read before the Odontological Society of Great Britain, published in the British Journal of Dental Science and copied in the Dental Cosmos, for April, 1874, pages 212 and 213.

I deem it unnecessary to quote from this paper. If the reader chooses to take the pains to examine he will be struck with the veri-similitude existing between the thoughts and the language of both authors on this subject.

I entertain a high and favorable opinion of Dr. Dunbar, and believe that he wrote this article with the best intentions.

It is to be regretted that he failed to exercise that thought and care which the matter demands. I say again, therefore, let no one be misled therewith, and believe that, by adopting Cumming's original method of working rubber, he will thereby evade the liability of infringement.

*Chillicothe, Oct.* 15, 1874. F. H. Rehwinkle.

